# Application of Machine Learning Algorithms in Predicting Major Adverse Cardiovascular Events after Percutaneous Coronary Intervention in Patients with New-Onset ST-Segment Elevation Myocardial Infarction

**DOI:** 10.31083/RCM25758

**Published:** 2025-02-21

**Authors:** Min Chen, Cuiling Sun, Li Yang, Ting Zhang, Jing Zhang, Chunli Chen

**Affiliations:** ^1^Department of Cardiology, The Second People’s Hospital of Hefei, Hefei Hospital Affiliated to Anhui Medical University, 230011 Hefei, Anhui, China; ^2^School of Nursing, Bengbu Medical University, 233030 Bengbu, Anhui, China; ^3^Department of Nursing, The Second People’s Hospital of Hefei, Hefei Hospital Affiliated to Anhui Medical University, 230011 Hefei, Anhui, China; ^4^The Fifth Clinical School of Medicine, Anhui Medical University, 230032 Hefei, Anhui, China

**Keywords:** new-onset STEMI, PCI, major adverse cardiovascular events, machine learning, SHAP analysis

## Abstract

**Background:**

This study aimed to develop and validate a predictive model for major adverse cardiovascular events (MACE) following percutaneous coronary intervention (PCI) in patients with new-onset ST-segment elevation myocardial infarction (STEMI) using four machine learning (ML) algorithms.

**Methods:**

Data from 250 new-onset STEMI patients were retrospectively collected. Feature selection was performed using the Boruta algorithm. Four ML algorithms—K-nearest neighbors (KNN), support vector machine (SVM), Complement Naive Bayes (CNB), and logistic regression—were applied to predict MACE risk. Model performance was evaluated using area under the curve (AUC), sensitivity, and specificity. Shapley Additive Explanations (SHAP) analysis was used to rank feature importance, and a nomogram was constructed for risk visualization.

**Results:**

Logistic regression showed the best performance (AUC = 0.814 in training, 0.776 in validation) compared to KNN, SVM, and CNB. SHAP analysis identified seven key predictors, including Killip classification, Gensini score, blood urea nitrogen (BUN), heart rate (HR), creatinine (CR), glutamine transferase (GLT), and platelet count (PCT). The nomogram provided accurate risk predictions with strong agreement between predicted and observed outcomes.

**Conclusions:**

The logistic regression model effectively predicts MACE risk after PCI in STEMI patients. The nomogram serves as a practical tool for clinicians, supporting personalized risk assessment and improving clinical decision-making.

## 1. Introduction

Acute myocardial infarction (AMI) is the most common type of cardiovascular disease in clinical practice, characterized by rapid progression, and a high mortality rate [1]. Its pathogenesis is primarily based on atherosclerotic lesions in the coronary arteries, leading to plaque rupture, platelet aggregation, and thrombosis, which ultimately cause prolonged and severe ischemic necrosis of the myocardium, and in severe cases, may endanger the patient’s life [2]. Percutaneous coronary intervention (PCI) has become the most widely used therapeutic approach in clinical practice because it can safely, rapidly, and efficiently help AMI patients restore coronary perfusion and alleviate acute-phase symptoms. However, studies have indicated that while PCI provides significant benefits, patients remain at risk of major adverse cardiovascular events (MACE) such as recurrent AMI, heart failure, malignant arrhythmias, and sudden cardiac death post PCI [3], with incidence rates as high as 27% [4]. The occurrence of MACE not only reduces the patient’s quality of life and increases their economic and social burdens, but also severely impacts their long-term prognosis and well-being [5]. Although current studies on MACE risk assessment in AMI patients have made some progress [6, 7], methodological limitations prevent a comprehensive understanding of the complex mechanisms and the strength of associations between clinical data, thus limiting the clinical applicability of these findings. In recent years, the rapid development of machine learning technology and its application in clinical data analysis have gained significant attention from medical institutions and researchers for their accurate and efficient predictive performance and clinical decision-making. By comprehensively analyzing large volumes of clinical and biochemical data, machine learning technology identifies risk factors that may be overlooked by traditional methods and plays a crucial role in guiding healthcare professionals to conduct more accurate risk assessments and make informed clinical decisions [8]. According to the results of previous studies, machine learning models have unlimited potential and value in dealing with complex and high-dimensional cardiovascular disease data and postoperative complication risk prediction [9]. It can help doctors better understand patients’ individualized risk of disease and adjust their treatment strategies accordingly, which can better optimize a patient’s long-term treatment outcomes [10].

In this study, we collected clinical data from patients with ST-segment elevation myocardial infarction (STEMI) admitted to the Department of Cardiology of a tertiary care hospital in Anhui Province, China, and constructed a risk prediction model for MACE in STEMI patients with the help of advanced machine learning algorithms, to provide effective assessment tools and methodological references to help healthcare professionals understand the risk of the disease in more depth and to formulate personalized scientific management strategies.

## 2. Materials and Methods

### 2.1 Data Source

This study retrospectively collected clinical data on 250 patients with a STEMI admitted to a tertiary general hospital in Anhui Province from June 2018 to December 2023, including patients’ demographic characteristics, laboratory tests, cardiac ultrasound, PCI data, MACE and other related information, with the specific variables and abbreviations, as shown in Appendix Table 5. This study was approved by the hospital ethics committee and all participants signed an informed consent form.

### 2.2 Study Participants

**Fig. 1.**
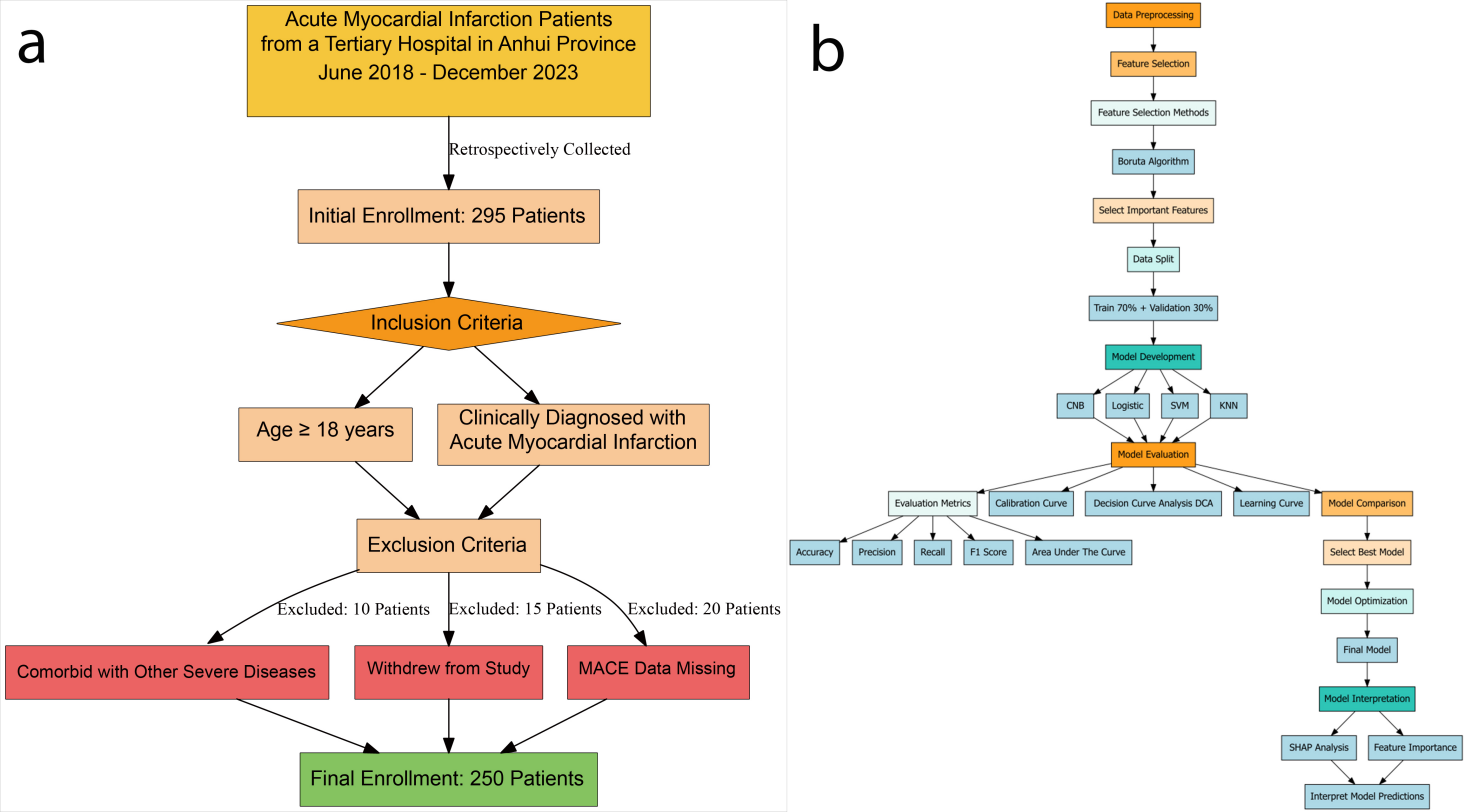
**Patients selection process and machine learning pipeline for acute myocardial infarction study**. (a) Data inclusion and exclusion flowchart. (b) Flowchart of ML data processing. Abbreviations: CNB, Complement Naive Bayes; KNN, K-nearest neighbors; Logistic, logistic regression; SVM, support vector machine; SHAP, Shapley Additive Explanations; MACE, major adverse cardiovascular events; ML, machine learning.

The study population consisted of patients who experienced a first time new-onset STEMI during the study period and underwent emergency PCI. STEMI diagnosis was made according to the Fourth Universal Definition of Myocardial Infarction (2018) and/or the guidelines of the European Society of Cardiology (ESC)/American College of Cardiology (ACC)/American Heart Association (AHA), including symptoms of chest pain and significant ST-segment elevation on the electrocardiogram (ECG) [11]. Inclusion criteria were as follows: (1) patients diagnosed with STEMI based on the aforementioned guidelines, including those with typical chest pain symptoms and ST-segment elevation on ECG; (2) patients undergoing emergency PCI, including successful revascularization; (3) aged ≥18 years; (4) in a clear state of mind and with good verbal communication; (5) patients who provided informed consent and cooperated with the study. Exclusion criteria were: (1) patients with acute infections, inflammatory diseases, or other major conditions (e.g., advanced cancer, end-stage renal disease) or a short prognosis of survival; (2) patients with a history of prior myocardial infarction or those who had experienced previous MACE; (3) patients with incomplete clinical data, such as missing medical records or postoperative follow-up data. The inclusion and exclusion process is illustrated in Fig. 1a.

### 2.3 MACE Event Types

MACE Types: Given the hemodynamic instability typical of acute STEMI patients, this study placed particular emphasis on perioperative MACE, especially within the first 7 days post-PCI. Patients were followed in real time during the hospitalization, with special attention to the immediate post-operative period. MACE was defined as the occurrence of at least one of the following adverse cardiovascular events [12]: (1) Myocardial infarction: defined as a new or recurrent myocardial infarction occurring after the procedure. (2) Coronary revascularization: includes repeat PCI or coronary artery bypass grafting (CABG) for recurrent cardiovascular issues. This specifically excludes planned elective revascularization procedures. (3) Cardiovascular death: includes all deaths due to cardiovascular causes, such as cardiac arrest and cardiogenic shock. (4) Stroke: refers to new or recurrent stroke occurring after surgery. (5) Hospitalized heart failure: hospitalization due to exacerbation of heart failure. (6) Malignant arrhythmia: including ventricular tachycardia (VT), ventricular fibrillation (VF) and other serious arrhythmias.

### 2.4 Statistical Analysis Methods

#### 2.4.1 Data Preprocessing

Data preprocessing is the first and most crucial step in creating machine learning (ML) models. In this study, the fine-grained preprocessing process of the raw dataset includes data import, handling missing values and outliers, coding the categorized data, and splitting the dataset into a training set and a validation set. In terms of dealing with missing values and outliers, this study initially cleansed and organized the raw data by deleting specific empty rows or filling in the data. Next, the collated datasets were split by randomization according to the 7:3 ratio, and they were sequentially divided into a training cohort (N = 175) and an internal validation cohort (N = 75).

#### 2.4.2 Statistical Analysis

Continuous variable information was described using mean ± standard deviation (±S), and categorical information was expressed as frequency and percentage n (%). For continuous variable information that conforms to normal distribution, One-way analysis of variance (ANOVA) or Fisher’s exact probability method was used to test the difference, those that did not conform to normal distribution were statistically analyzed using the Mann-Whitney U test, and discrete categorical variables were compared using Chi-square test to compare the difference. ML algorithms tend to perform better than traditional analytical methods in predicting the results of large datasets [13]. In this study, four ML algorithms, namely K-nearest neighbors (KNN), support vector machine (SVM), Complement Naive Bayes (CNB), and logistic regression (Logistic), were used to construct a prediction model for the occurrence of MACE after PCI in patients with an acute STEMI, and based on the area under the curve (AUC) value, accuracy, sensitivity, specificity, positive predictive value, negative predictive value, and F1 score, the performance of each model was compared and evaluated. The area under the receiver operating characteristic (ROC) curve was used to assess the model’s classification ability, the accuracy of the model’s predictive probability was reflected by the calibration curve, the accuracy of the model’s classification was visualized with the help of the number of true positives, false positives, true negatives, and false negatives provided by the confusion matrix. The decision curve (DCA) was used to assess the net benefit of the different models across multiple clinical thresholds. The learning curve revealed how well the model over- or under-fitted the data, reflecting the mean square error of the model with respect to the training set and the validation set. When comparing the performance of ML algorithms, an AUC closer to 1 shows superior classification model performance. After screening the best ML model by comparing multiple models, the model was fine-tuned. Shapley Additive Explanations (SHAP) analysis and feature ranking are applied to interpret the best model. Finally, the risk factors screened by the optimized algorithm are extracted and plotted in a nomogram. All statistical analyses in this study were conducted using Python (version 3.11.4, Python Software Foundation, Wilmington, DE, USA) and R (version 4.2.3, The R Foundation for Statistical Computing, Vienna, Austria). A *p*-value of <0.05 was considered statistically significant. The detailed data analysis workflow is illustrated in Fig. 1b.

## 3. Results

### 3.1 Baseline Characteristics

A total of 250 participants were enrolled in this study for model construction, and patients with a STEMI undergoing PCI were randomly assigned to 2 cohorts, the training set (N = 175) and the validation set (N = 75). The number of cases with MACE that occurred in the training set was 51 cases, or 29.1%, and in the validation set, the number of cases with MACE occurred in 17 cases, or 22.7%. The demographic, laboratory tests, hemodynamics, PCI intraoperative data and other baseline characteristics of the two groups are shown in Table 1. As shown in Table 1, except for the diastolic blood pressure (BP) of the patients in the validation cohort, which was higher than that of the training cohort and was significantly difference (*p* < 0.05), the two groups of patients had an overall balanced distribution in all other aspects, and the difference was not statistically significant (*p* > 0.05), which indicated that the baseline data were comparable.

**Table 1.** **Baseline information of participants**.

Variable		Overall, N = 250	Training, N = 175	Validation, N = 75	*p*-value
MACE, n (%)		68 (27)	51 (29)	17 (23)	0.292
Sex, n (%)		202 (81)	138 (79)	64 (85)	0.234
Age, median (IQR)		62.500 (53.000–73.500)	63.000 (53.500–73.000)	60.000 (52.500–73.000)	0.631
Killip, n (%)					0.666
	I	192 (77)	131 (75)	61 (81)	
	II	34 (14)	25 (14)	9 (12)	
	III	13 (5.2)	11 (6.3)	2 (2.7)	
	IV	11 (4.4)	8 (4.6)	3 (4.0)	
SP, median (IQR)		124.000 (110.000–140.000)	124.000 (106.000–138.000)	127.000 (114.500–140.000)	0.075
BP, median (IQR)		72.000 (65.000–82.000)	70.000 (64.000–80.000)	77.000 (70.000–88.000)	0.008
HR, median (IQR)		78.000 (68.000–86.000)	78.000 (68.000–84.500)	78.000 (71.500–90.000)	0.102
Smoking, n (%)		152 (61)	106 (61)	46 (61)	0.910
TDM, n (%)		66 (26)	47 (27)	19 (25)	0.802
HP, n (%)		141 (56)	100 (57)	41 (55)	0.717
Lesion location, n (%)					0.918
	0	3 (1.2)	2 (1.1)	1 (1.3)	
	1	112 (45)	76 (43)	36 (48)	
	2	36 (14)	26 (15)	10 (13)	
	3	99 (40)	71 (41)	28 (37)	
Number of vessels diseased, n (%)					0.535
	0	2 (0.8)	1 (0.6)	1 (1.3)	
	1	77 (31)	57 (33)	20 (27)	
	2	77 (31)	55 (31)	22 (29)	
	3	94 (38)	62 (35)	32 (43)	
Gensini, median (IQR)		61.500 (42.625–85.750)	60.000 (42.000–86.500)	66.000 (51.500–84.500)	0.292
Thrombolysis, n (%)		62 (25)	48 (27)	14 (19)	0.142
Bivalirudin trifluoroacetate salt, n (%)		44 (18)	34 (19)	10 (13)	0.246
Number of stents, n (%)		220 (88)	151 (86)	69 (92)	0.203
WBC, median (IQR)		10.085 (7.863–12.348)	10.140 (7.865–12.335)	9.450 (7.815–12.470)	0.566
N, median (IQR)		7.510 (5.595–9.970)	7.670 (5.870–9.970)	7.070 (5.340–9.900)	0.433
L, median (IQR)		1.365 (0.993–1.968)	1.340 (0.990–1.950)	1.500 (1.025–1.975)	0.313
M, median (IQR)		0.565 (0.400–0.800)	0.580 (0.400–0.800)	0.500 (0.400–0.780)	0.513
RBC, median (IQR)		4.505 (4.003–4.888)	4.420 (3.960–4.860)	4.630 (4.170–4.985)	0.192
HB, median (IQR)		136.800 (124.000–151.000)	136.000 (124.000–151.000)	139.000 (125.500–150.500)	0.593
PLT, median (IQR)		197.500 (156.125–239.750)	201.000 (159.000–241.000)	195.000 (154.550–235.500)	0.743
MPV, median (IQR)		10.570 (9.700–11.400)	10.500 (9.650–11.350)	10.600 (9.750–11.600)	0.678
PCW, median (IQR)		16.200 (13.325–16.600)	16.200 (13.450–16.565)	16.200 (13.250–16.700)	0.412
PCT, median (IQR)		0.210 (0.170–0.248)	0.210 (0.170–0.250)	0.201 (0.171–0.244)	0.494
GLU, median (IQR)		6.310 (5.455–8.138)	6.380 (5.560–8.280)	5.990 (5.345–7.695)	0.234
BUN, median (IQR)		5.435 (4.330–6.908)	5.600 (4.320–6.975)	5.230 (4.340–6.850)	0.496
CR, median (IQR)		71.000 (59.400–83.000)	73.000 (59.000–85.200)	68.000 (61.300–79.750)	0.288
UA, median (IQR)		360.450 (296.000–434.925)	355.000 (286.650–433.400)	365.000 (312.300–438.950)	0.375
Total protein, median (IQR)		62.050 (59.200–65.875)	62.000 (58.850–66.000)	62.200 (59.700–65.550)	0.522
DBIL, median (IQR)		5.000 (3.900–6.500)	5.100 (3.900–6.300)	4.950 (4.000–7.275)	0.663
IBIL, median (IQR)		13.200 (9.900–17.275)	12.900 (10.000–17.000)	13.500 (9.250–17.350)	0.933
GLT, median (IQR)		43.000 (27.000–71.000)	43.000 (28.000–71.000)	42.000 (24.500–75.500)	0.886
GST, median (IQR)		166.500 (87.000–289.250)	163.000 (89.500–292.500)	174.000 (86.000–286.000)	0.919
TG, median (IQR)		1.495 (1.045–2.138)	1.520 (1.065–2.075)	1.420 (1.015–2.295)	0.881
TC, median (IQR)		4.250 (3.723–4.985)	4.290 (3.730–5.070)	4.250 (3.700–4.860)	0.568
HDL, median (IQR)		1.065 (0.910–1.218)	1.060 (0.905–1.200)	1.080 (0.910–1.250)	0.775
LDL, median (IQR)		2.665 (2.205–3.310)	2.680 (2.255–3.395)	2.560 (2.170–3.260)	0.276
VLDL, median (IQR)		0.300 (0.210–0.420)	0.300 (0.210–0.400)	0.280 (0.200–0.450)	0.784
LVD, median (IQR)		47.000 (43.250–51.000)	47.000 (43.500–51.000)	47.000 (43.500–51.000)	0.961

Abbreviations: MACE, major adverse cardiovascular events; SP, systolic blood pressure; BP, diastolic blood pressure; HR, heart rate; TDM, type 2 diabetes mellitus; HP, high blood pressure; Gensini, coronary artery lesion stenosis score; WBC, white blood cell; N, absolute neutrophil value; L, lymphocyte absolute value; M, absolute monocyte value; RBC, red blood cell; HB, hemoglobin; PLT, platelet count; MPV, mean platelet volume; PCW, platelet volume distribution width; PCT, plateletcrit; GLU, glucose; BUN, blood urea nitrogen; CR, creatinine; UA, uric acid; DBIL, direct bilirubin; IBIL, indirect bilirubin; GLT, glutamine transferase; GST, glutathione S-transferase; TG, triglyceride; TC, total cholesterol; HDL, high-density lipoprotein; LDL, low-density lipoprotein;VLDL, very low-density lipoprotein; LVD, left ventricular internal diameter; IQR, interquartile range.

### 3.2 Model Construction and Performance Evaluation

#### 3.2.1 Feature Variable Selection

**Fig. 2.**
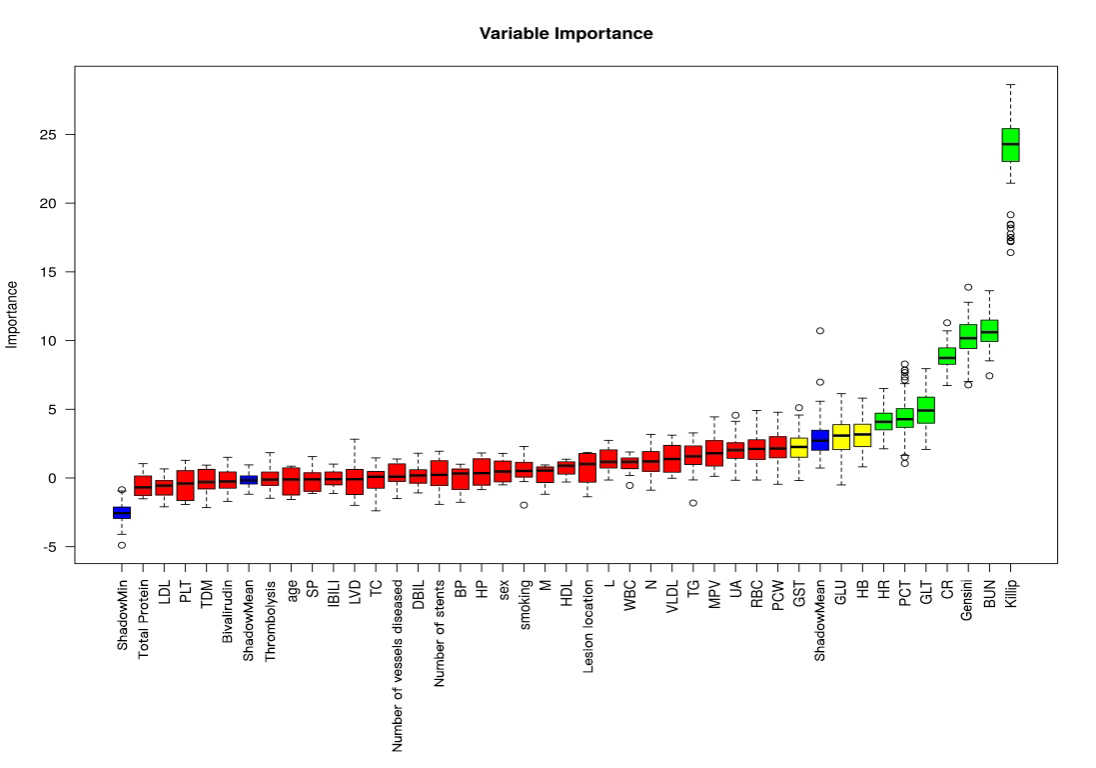
**Results of feature variable selection based on Boruta’s algorithm**. (Note: where Tentative variables are labeled yellow, Rejected is red, Accepted is green, and Shadow is blue). Abbreviations: BUN, blood urea nitrogen; Gensini, coronary artery lesion stenosis score; CR, creatinine; GLT, glutamine transferase; PCT, plateletcrit; HR, heart rate; HB, hemoglobin; GLU, glucose; GST, glutathione S-transferase; PCW, platelet volume distribution width; RBC, red blood cell; UA, uric acid; MPV, mean platelet volume; TG, triglyceride; VLDL, very low-density lipoprotein; N, absolute neutrophil value; WBC, white blood cell; L, lymphocyte absolute value; HDL, high-density lipoprotein; M, absolute monocyte value; HP, high blood pressure; BP, diastolic blood pressure; DBIL, direct bilirubin; TC, total cholesterol; LVD, left ventricular internal diameter; IBIL, indirect bilirubin; SP, systolic blood pressure; TDM, type 2 diabetes mellitus; PLT, platelet count; LDL, low-density lipoprotein.

A total of 40 variables were included in this study. In order to improve the accuracy of the model, we screened the variables for features, by using the Boruta algorithm to select the feature variables for the 40 risk factors for the occurrence of MACE after surgery in patients with acute ST-segment elevation myocardial infarction. Finally 7 variables were included into the model, the specific screening results are shown in Fig. 2.

#### 3.2.2 Multi-Model Comparison

In this study, we employed four ML algorithms (KNN, SVM, CNB, and Logistic) to construct predictive models for the risk of MACE after PCI in patients with STEMI. Model performance was comprehensively evaluated using AUC values, accuracy, sensitivity, specificity, positive predictive values, negative predictive values, and F1 score.

We utilized nested cross-validation to optimize model hyperparameters and evaluate model performance. Specifically, we used 5-fold outer cross-validation for model evaluation, and within each fold, we performed 3-fold inner cross-validation for hyperparameter tuning. This approach ensures a more robust estimate of model performance and reduces the risk of overfitting.

The results of the comparison of the predictive performance of each model in the training and validation sets are shown in Tables 2,3, respectively. From Table 2, it can be seen that the model that demonstrated the best classification performance in the training set was the Logistic model (AUC = 0.814 ± 0.006), followed by the KNN model (AUC = 0.805 ± 0.028). The Logistic model also exhibited the highest sensitivity (0.618 ± 0.066) and F1 score (0.632 ± 0.016) in the training set.

**Table 2.** **Comparison of the prediction performance of different models in the training set**.

Classification model	AUC (SD)	Accuracy (SD)	(level of) Sensitivity (SD)	Specificity (SD)	Positive predictive value (SD)	Negative predictive value (SD)	F1 score (SD)
KNN	0.805 (0.028)	0.800 (0.021)	0.346 (0.040)	0.970 (0.017)	0.814 (0.095)	0.799 (0.012)	0.484 (0.053)
CNB	0.572 (0.029)	0.678 (0.031)	0.470 (0.081)	0.755 (0.070)	0.428 (0.046)	0.793 (0.011)	0.440 (0.026)
SVM	0.720 (0.027)	0.782 (0.020)	0.544 (0.096)	0.871 (0.058)	0.632 (0.076)	0.838 (0.019)	0.572 (0.037)
Logistic	0.814 (0.006)	0.804 (0.024)	0.618 (0.066)	0.874 (0.055)	0.661 (0.069)	0.861 (0.014)	0.632 (0.016)

KNN, K-nearest neighbors; CNB, Complement Naive Bayes; SVM, support vector machine; SD, standard deviation; AUC, area under the curve; Logistic, logistic regression.

**Table 3.** **Comparison of the prediction performance of different models in the validation set**.

Classification model	AUC (SD)	Accuracy (SD)	(level of) Sensitivity (SD)	Specificity (SD)	Positive predictive value (SD)	Negative predictive value (SD)	F1 score (SD)
KNN	0.641 (0.070)	0.756 (0.039)	0.193 (0.103)	0.967 (0.011)	0.637 (0.188)	0.763 (0.031)	0.292 (0.143)
CNB	0.533 (0.104)	0.636 (0.034)	0.385 (0.127)	0.732 (0.085)	0.352 (0.056)	0.763 (0.028)	0.357 (0.058)
SVM	0.685 (0.090)	0.732 (0.055)	0.471 (0.062)	0.830 (0.076)	0.539 (0.168)	0.807 (0.022)	0.493 (0.078)
Logistic	0.776 (0.038)	0.784 (0.081)	0.614 (0.149)	0.845 (0.163)	0.702 (0.196)	0.861 (0.029)	0.615 (0.047)

KNN, K-nearest neighbors; CNB, Complement Naive Bayes; SVM, support vector machine; SD, standard deviation; AUC, area under the curve; Logistic, logistic regression.

In the validation set (Table 3), the Logistic model demonstrated the highest classification performance, achieving an AUC of 0.776 ± 0.038. This was followed by the SVM model, which had an AUC of 0.685 ± 0.090. Additionally, the Logistic model outperformed others in sensitivity (0.614 ± 0.149) and F1 score (0.615 ± 0.047), maintaining its status as the best-performing model in the validation set.

**Fig. 3.**
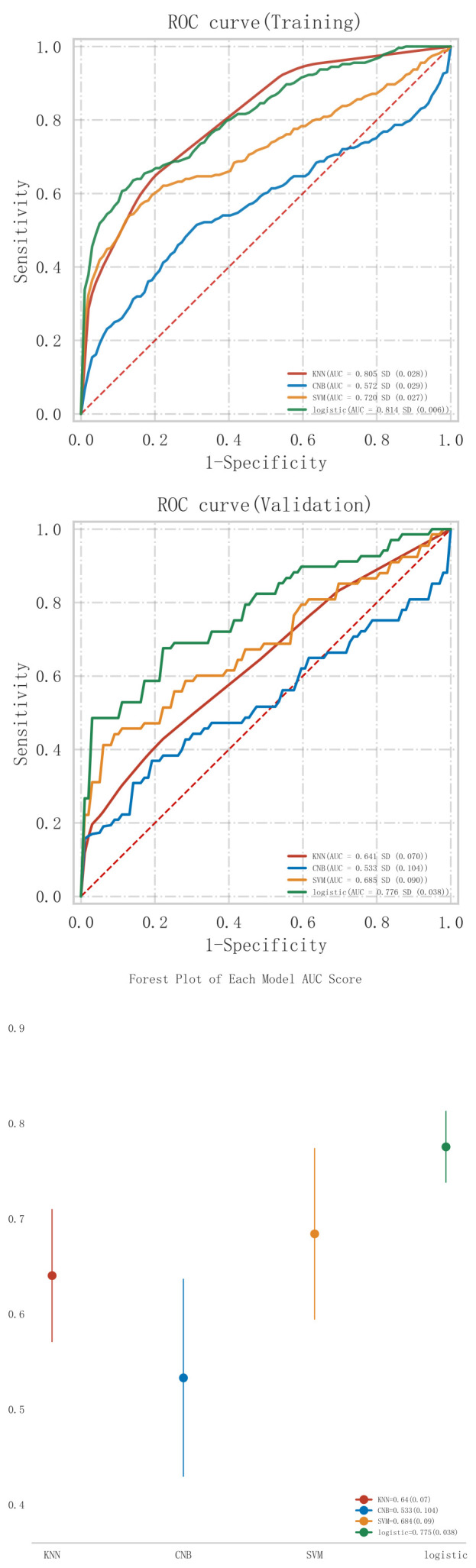
**ROC curve analysis and forest plot of ML algorithms for predicting the occurrence of MACE after PCI in patients with acute myocardial infarction**. ROC, receiver operating characteristic; ML, machine learning; MACE, major adverse cardiovascular events; PCI, percutaneous coronary intervention; KNN, K-nearest neighbors; CNB, Complement Naive Bayes; Logistic, logistic regression; SVM, support vector machine; AUC, area under the curve.

**Fig. 4.**
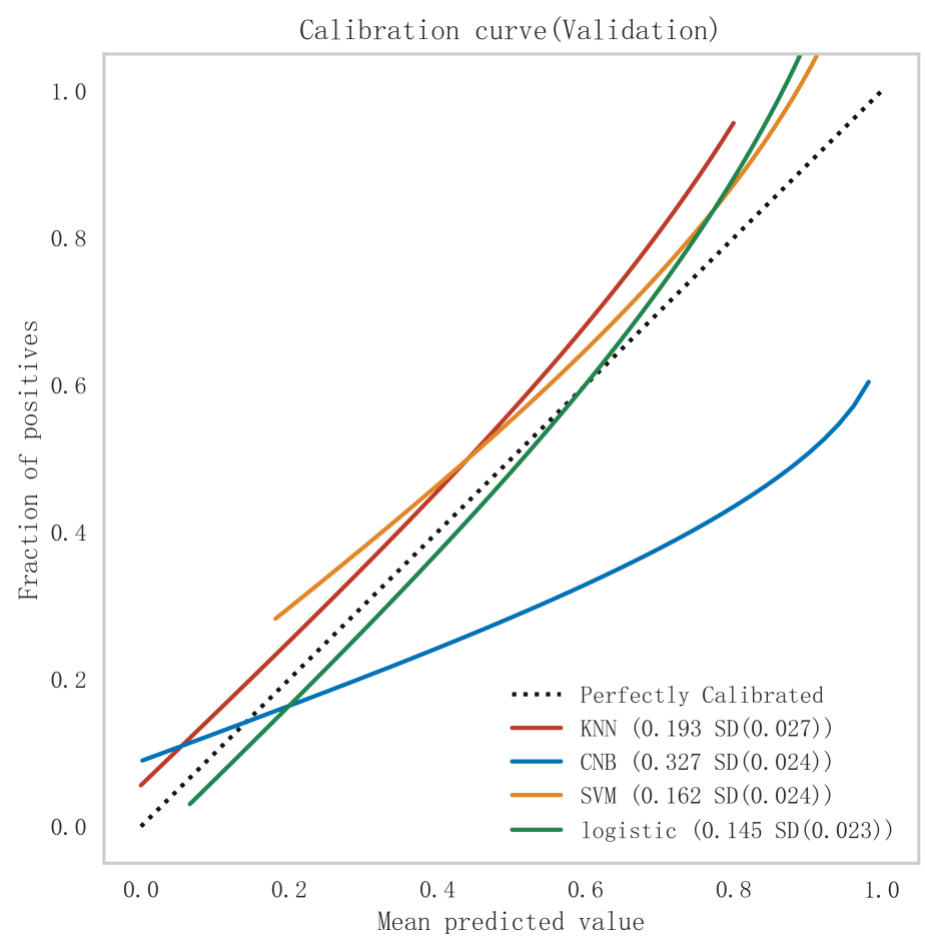
**Comparison of calibration curves for each model in the validation set**. KNN, K-nearest neighbors; CNB, Complement Naive Bayes; Logistic, logistic regression; SVM, support vector machine.

**Fig. 5.**
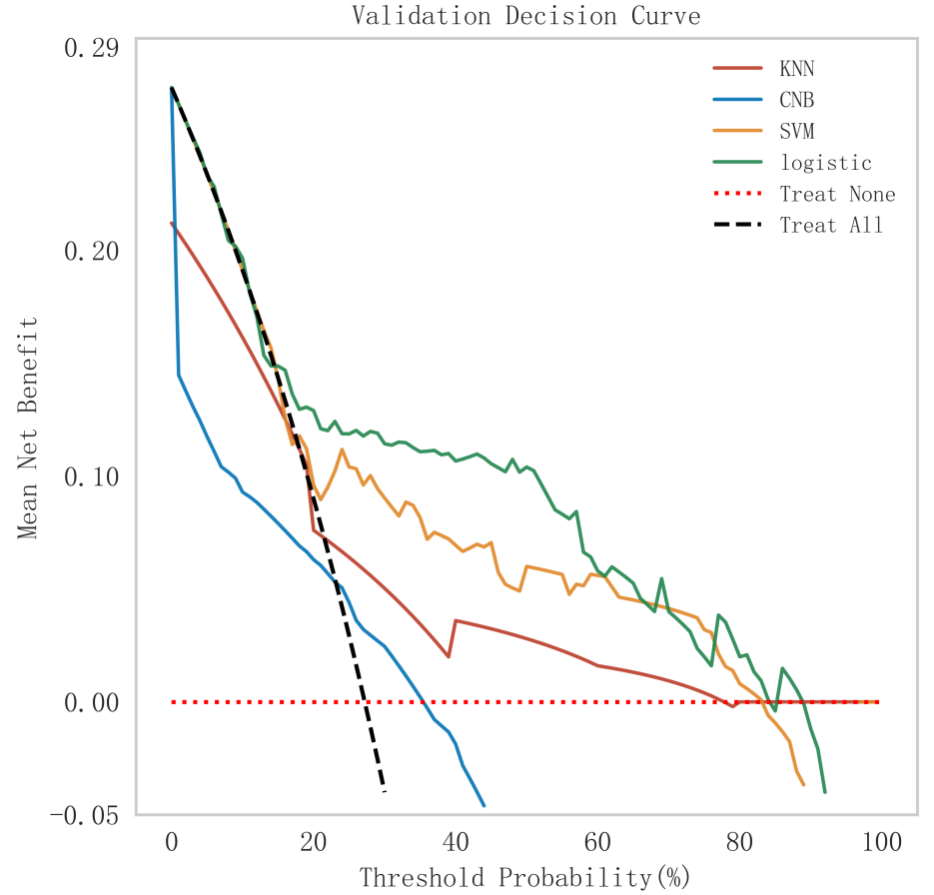
**Analysis of clinical decision curves for each model in the validation set**. KNN, K-nearest neighbors; CNB, Complement Naive Bayes; Logistic, logistic regression; SVM, support vector machine.

Among all the models constructed, the Logistic model performed best in terms of key metrics such as AUC, sensitivity, and F1 score in both cohorts of the training and validation sets, and showed good stability. Notably, the performance of the Logistic model in the validation set surpassed other models, indicating better generalization capability. Among all the models constructed, the Logistic model performed best in terms of key metrics such as AUC, sensitivity, and F1 score in both cohorts of the training and validation sets, and showed good stability. Notably, the performance of the Logistic model in the validation set surpassed other models, indicating better generalization capability. In contrast, the Logistic model performed more prominently in terms of clinical discrimination (see Fig. 3), model fit (see Fig. 4), and clinical applicability (as shown in Fig. 5) in both cohorts of the training and validation sets, further demonstrating its robustness.

To further evaluate the statistical significance of the differences in AUC values between models, we conducted the Delong test. The results of the Delong test, as shown in Table 4, indicate that although the Logistic model generally outperformed other models, the differences in AUC values between some models were not statistically significant. Specifically, the *p*-values between KNN and logistic regression (*p* = 0.209), as well as between SVM and logistic regression (*p* = 0.364), suggest that these differences were not statistically significant (*p* > 0.05). However, the comparison between CNB and logistic regression (*p* = 0.115) approached significance, highlighting the competitive performance of the Logistic model compared to other models.

**Table 4.** ***p*-values from delong test for pairwise AUC comparisons between machine learning models**.

Name	KNN	CNB	SVM	Logistic
KNN	NA	0.24	0.261	0.209
CNB	0.24	NA	0.437	0.115
SVM	0.261	0.437	NA	0.364
Logistic	0.209	0.115	0.364	NA

KNN, K-nearest neighbors; CNB, Complement Naive Bayes; SVM, support vector machine; Logistic, logistic regression; NA, not applicable.

Therefore, considering its consistent superior performance in both the training and validation sets, good generalization ability, and clinical interpretability, the logistic regression model was selected as the optimal model for this study.

#### 3.2.3 Establishment and Optimization of the Optimal Model

**Fig. 6.**
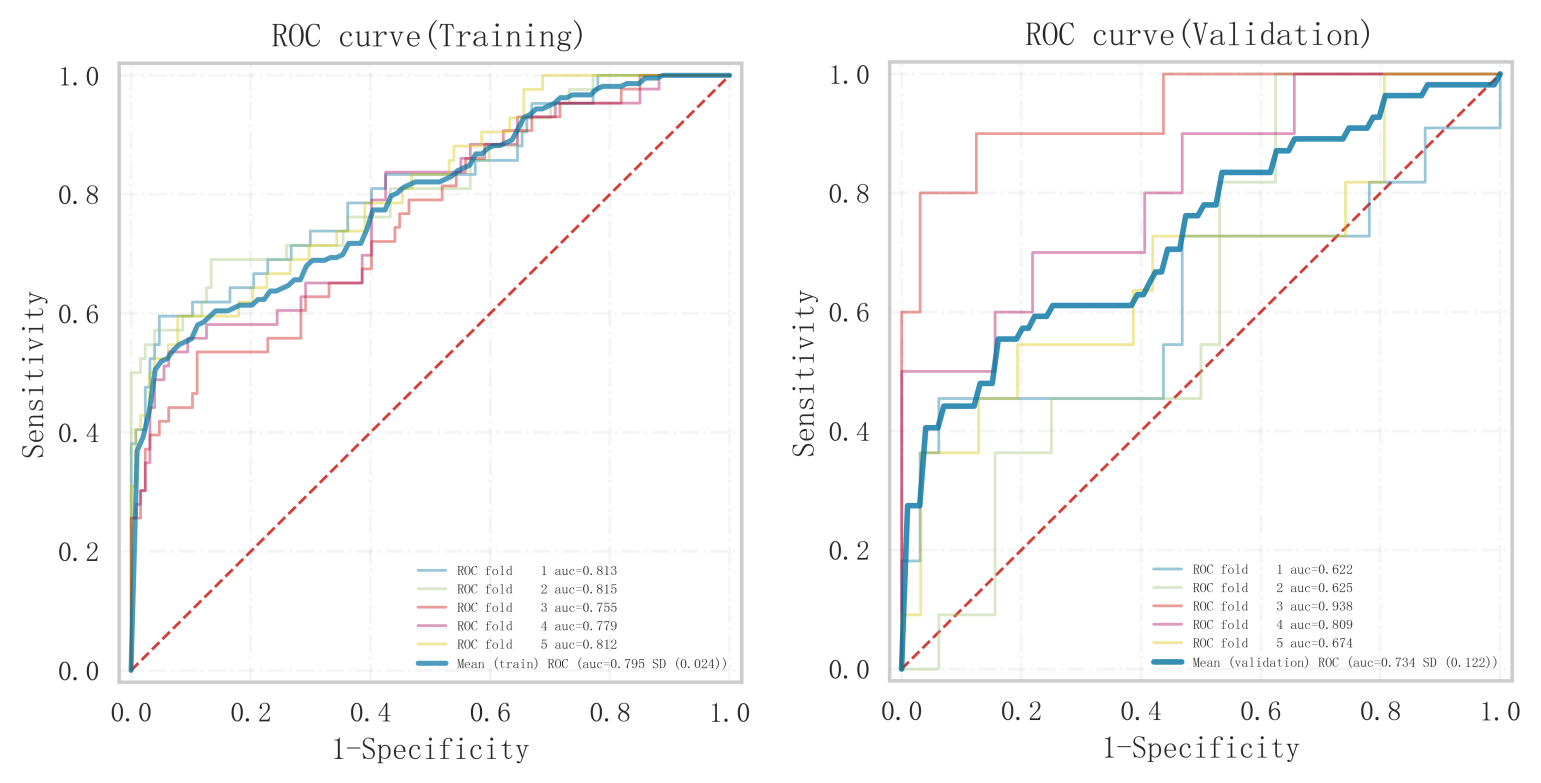
**Analysis of the area under the ROC curves of the training and validation sets under 5-fold cross-validation within the logistic regression model**. ROC, receiver operating characteristic.

**Fig. 7.**
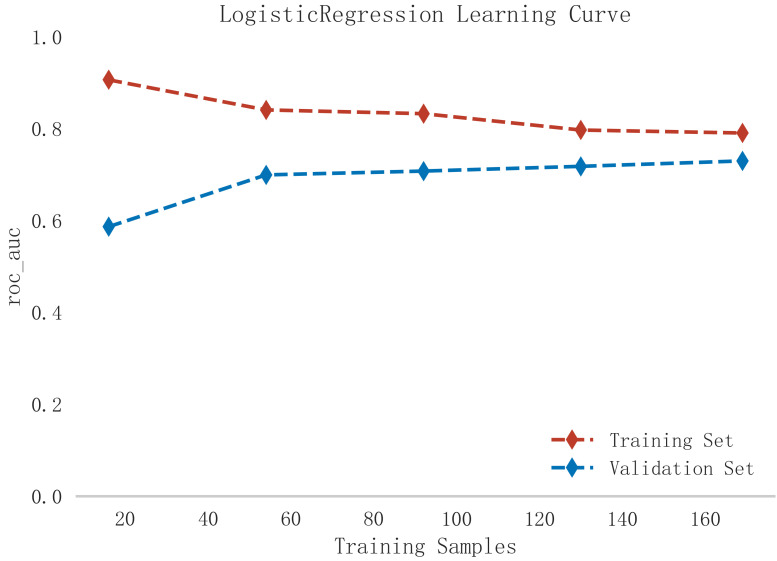
**Learning curve analysis of logistic regression model in training and validation sets**.

**Fig. 8.**
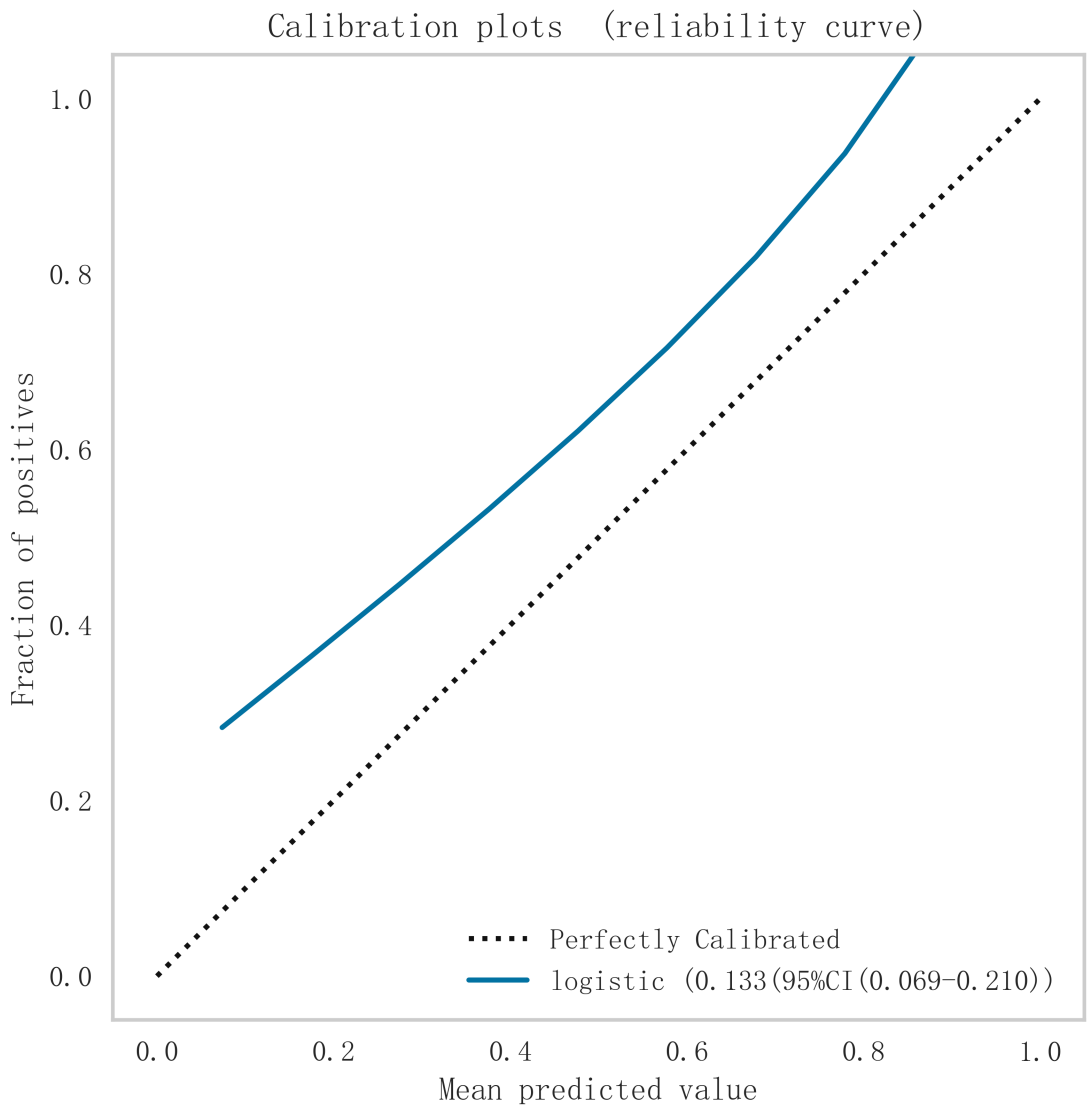
**Calibration curve analysis of logistic regression model**. Logistic, logistic regression.

**Fig. 9.**
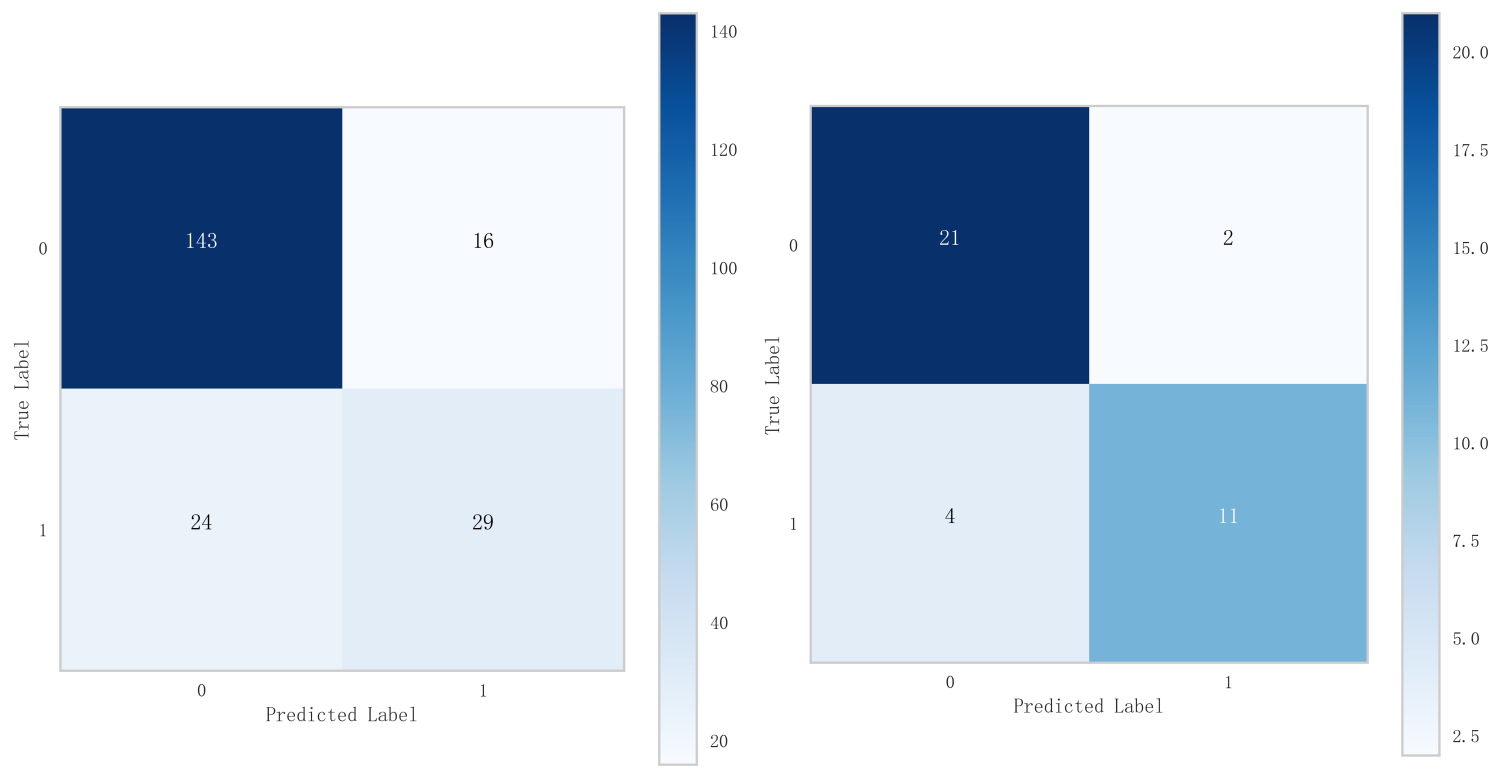
**Mixed matrix analysis of logistic regression model in training and validation sets**.

**Fig. 10.**
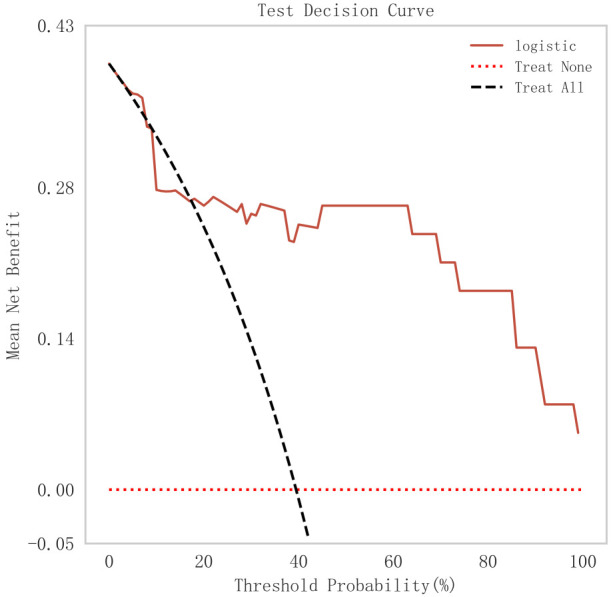
**DCA curve analysis of logistic regression model**. DCA, decision curve.

In summary, the logistic model was finally selected as the optimal model for predicting the occurrence of MACE after PCI in patients with STEMI in this study. In this study, 15% of the overall samples were randomly selected as the test set (N = 37), and the other remaining samples were used as the validation set (N = 213) to optimize the logistic regression model using internal 5-fold cross-validation. The results showed that the model had an AUC value of 0.795 for the area under the ROC curve in the test set, and 0.734 in the validation set, as shown in Fig. 6. Given that the performance of the validation set for the AUC metrics did not exceed the test set or exceeded the criterion that a ratio of less than 10% can be considered a successful model fit, this study determined that the logistic model was well suited for this dataset. In addition, learning curves were plotted in this study to assess whether there was any overfitting of the model. As can be seen in Fig. 7, the difference in error between the training set and the test set in this model decreases and stabilizes with the increase in the number of training samples, indicating that there is no overfitting or underfitting in this model. From the calibration curve (Fig. 8) and mixing matrix (Fig. 9) analysis in the figure, we showed that the logistic regression model has good accuracy and consistency in predicting the risk of MACE after PCI in patients with STEMI. Finally, we evaluated the clinical applicability and benefit level of the model through the clinical decision curve. The results of Fig. 10 showed that the logistic regression model could help patients achieve a better net clinical benefit at a lower threshold probability.

#### 3.2.4 Interpretation of the Model and Ranking of Significant Characteristic Variables

**Fig. 11.**
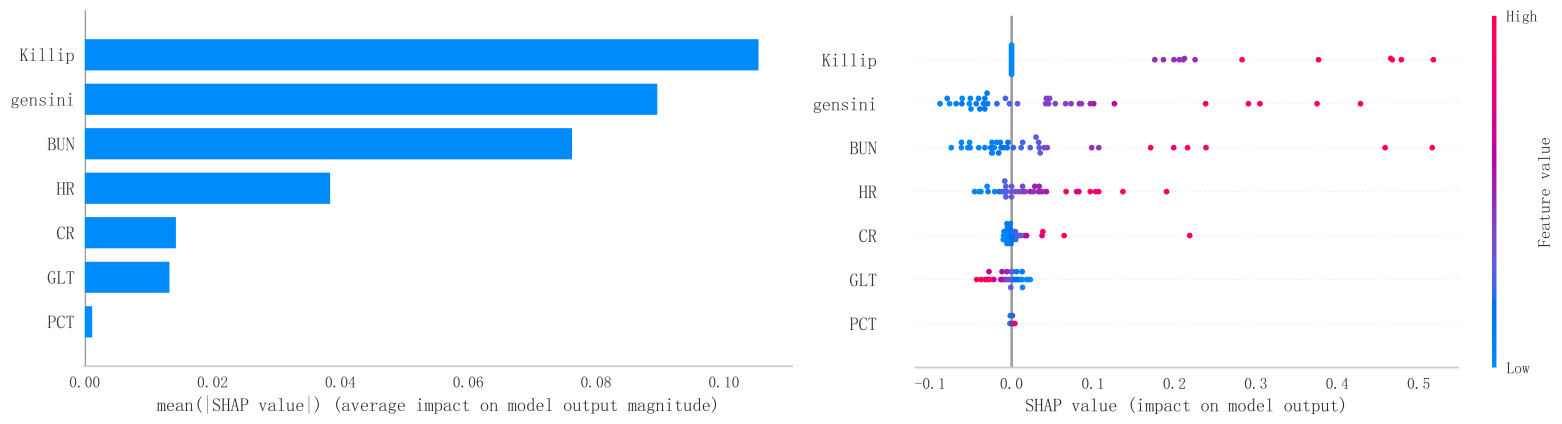
**The top 7 important eigenvariables and eigenvalues screened based on Boruta’s algorithm**. BUN, blood urea nitrogen; HR, heart rate; CR, creatinine; GLT, glutamine transferase; PCT, platelet count; SHAP, Shapley Additive Explanations.

In order to improve the precision and interpretability of the ML model, we also used the SHAP analysis method to interpret and rank the importance of the included variables to determine the magnitude of the contribution of each variable for the risk of developing MACE after PCI in the population of patients with a STEMI. The results of the feature variable selection and importance analysis based on Boruta’s algorithm are shown in Fig. 11. Fig. 11 shows the top 7 important feature variables and eigenvalues screened, which are Killip, Gensini, blood urea nitrogen (BUN), heart rate (HR), creatinine (CR), glutamine transferase (GLT), and platelet count (PCT).

#### 3.2.5 Establishment and Evaluation of the Nomogram

**Fig. 12.**
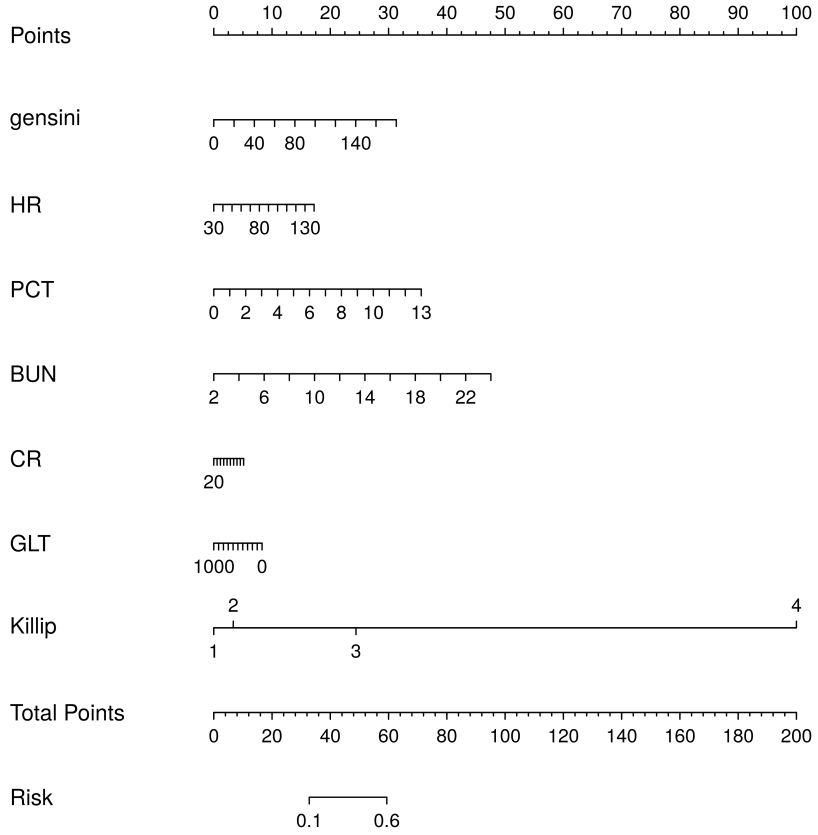
**Nomogram to show the risk prediction of postoperative MACE in patients with acute myocardial infarction**. BUN, blood urea nitrogen; HR, heart rate; CR, creatinine; GLT, glutamine transferase; PCT, platelet count; MACE, major adverse cardiovascular events.

**Fig. 13.**
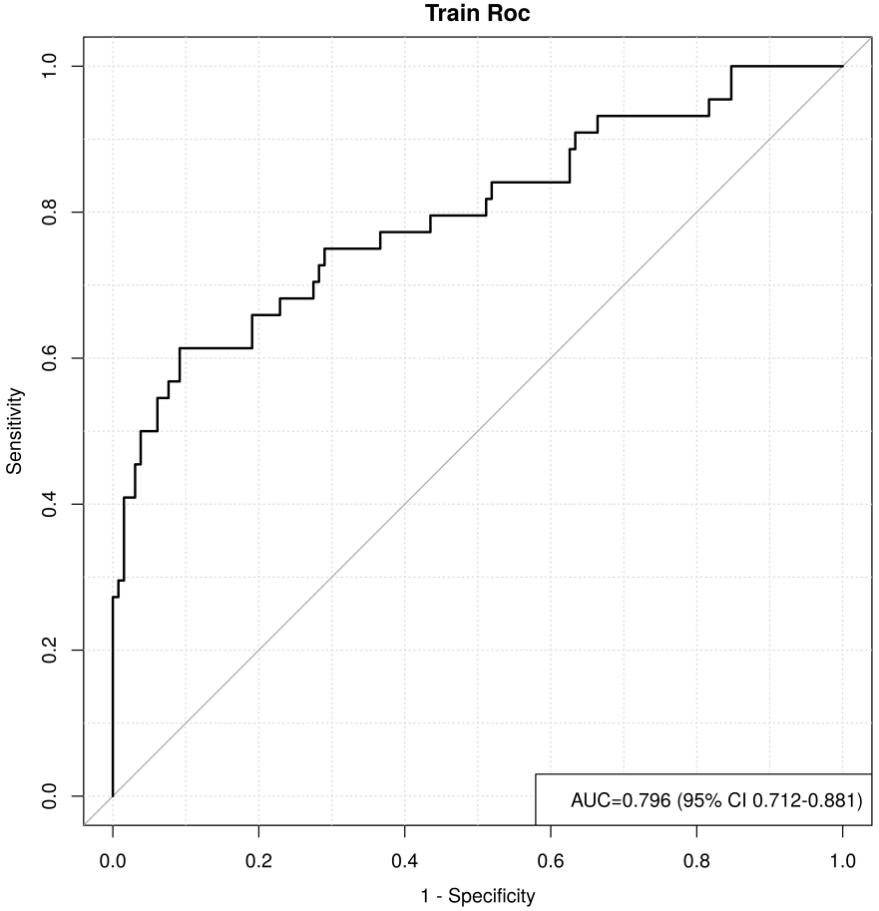
**ROC curve for the training set**. ROC, receiver operating characteristic; AUC, area under the curve; CI, confidence interval.

**Fig. 14.**
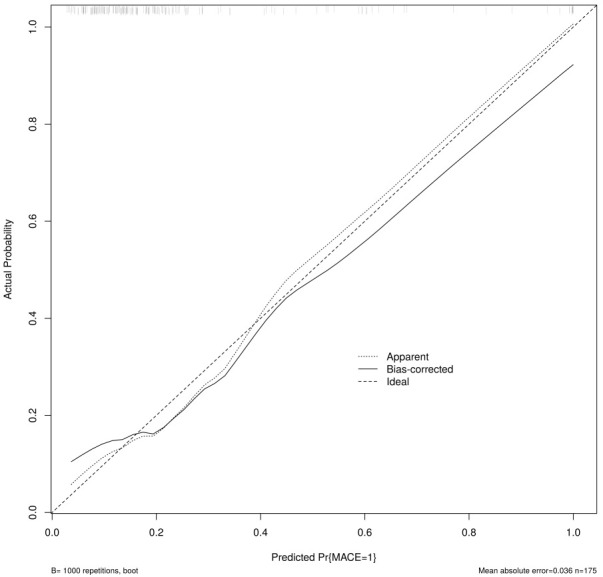
**Calibration curve of the nomogram for the training set**. MACE, major adverse cardiovascular events.

Combining the results of the above analyses, we found that Killip, Gensini, BUN, HR, CR, GLT, and PCT are independent risk factors for the occurrence of MACE after PCI in patients with an STEMI. In this study, we entered these 7 variables into the logistic regression model and drew the nomogram (Fig. 12). Compared with complex logistic regression formulas, nomograms are simple, straightforward, intuitively visualized, and often have better clinical utility. When used with a straightedge as a plumb line, the risk scores of each characteristic variable in the column chart are first calculated separately according to the specific conditions of the individual, and then these scores are all added together to obtain the total risk score, which can intuitively and clearly predict the risk probability of MACE after PCI in patients with an STEMI. The higher the total score, the greater the risk of MACE after PCI in patients with STEMI. The results of the analysis in Figs. 13,14 confirmed that the model has good clinical predictive value and accuracy.

To further enhance the clinical applicability and accessibility of our model, we have developed a dynamic version of the nomogram using the Shiny package in R. This interactive tool allows clinicians to input patient-specific data through a user-friendly interface, providing real-time prediction for the risk of MACE. The dynamic nomogram offers several advantages over the static version, including precise value input, instant risk calculation, and improved readability. It effectively addresses the issue of unclear risk lines that may be present in static nomograms. We have deployed this dynamic nomogram online, and it can be accessed at: [https://prediction1model.shinyapps.io/dynnomapp/].

## 4. Discussion

In this study, we employed four machine learning algorithms to conduct an in-depth analysis and modeling of the demographic, laboratory, and clinical characteristics of acute STEMI patients. Our goal was to accurately predict the risk of MACE following PCI in STEMI patients. After thoroughly assessing the importance of characteristic variables and comparing model performance, the logistic regression model proved to be the most effective in terms of discriminatory power, accuracy, and clinical applicability. By ranking feature importance, we identified seven key variables—Killip, Gensini, BUN, HR, CR, GLT, and PCT—that significantly impacted the prediction model. These variables are discussed in detail below.

Previous research has established that MACE after PCI is a significant contributor to prolonged hospital stays, increased healthcare costs, and elevated mortality risk in STEMI patients. This can significantly affect their long-term prognosis and health-related quality of life [14, 15]. Consequently, early identification of critical risk factors for MACE following PCI in STEMI patients, along with timely interventions, is crucial.

The Killip classification, an important indicator of heart failure severity, was identified in this study as a strong predictor of MACE. This aligns with the findings of Takasaki et al, who showed that higher Killip classifications were strongly correlated with both short- and long-term mortality, particularly in post-STEMI patients [16]. This correlation likely arises because heart failure is a direct result of diminished myocardial contractility, which is closely linked to elevated inflammatory markers, endothelial dysfunction, and cardiac remodeling. Furthermore, heart failure often coincides with reduced renal function, resulting in fluid retention and increased cardiac workload, thereby elevating the risk of adverse cardiac events [17]. Clinically, STEMI patients with high Killip classifications require more aggressive monitoring and interventions. This may involve the use of diuretics to manage fluid overload, as well as medications such as β-blockers and angiotensin converting enzyme (ACE) inhibitors to improve cardiac function. These measures are critical in reducing post-PCI adverse events and improving long-term patient outcomes.

The Gensini score, which quantifies the severity of coronary lesions based on the degree of stenosis and lesion location seen on coronary angiography, was also identified as an independent predictor of MACE in STEMI patients after a PCI. This finding is consistent with previous studies [18, 19]. The Gensini score provides valuable insights into the severity and prognosis of STEMI by accounting for the number, location, and extent of coronary lesions [20]. For patients with high Gensini scores, clinical teams should be proactive in adjusting treatment strategies, providing close follow-up care, and focusing on delaying the progression of coronary stenosis to improve therapeutic and rehabilitative outcomes.

Additionally, our study found that BUN levels were closely linked to the risk of MACE after PCI in STEMI patients. BUN, a byproduct of protein metabolism, reflects early cardiac and renal hemodynamic changes and serves as a strong predictor of poor cardiovascular outcomes, as supported by numerous studies [21, 22, 23]. Horiuchi *et al*. [21] also demonstrated that high BUN levels significantly increased the risk of MACE in STEMI patients post-PCI, leading to longer hospital stays and higher mortality rates.

HR, a common clinical marker of cardiac function, has received increasing attention for its predictive value in cardiovascular disease. A chronically elevated heart rate is a key risk factor for adverse cardiovascular events, heart failure, and longer hospitalizations [24, 25, 26, 27, 28]. Sympathetic hyperactivity and excessive catecholamine secretion are common causes of increased heart rate, which leads to higher intravascular pressure, damage to the coronary endothelium, and the promotion of inflammatory factors. These processes accelerate atherosclerosis, increase the risk of arrhythmias, and elevate the likelihood of recurrent myocardial infarction. Effective heart rate management through β-blockers and other medications can alleviate patient symptoms and reduce adverse events in STEMI patients.

Our study also revealed that CR, an indicator of renal function, was an important predictor of MACE following PCI in STEMI patients. This aligns with previous studies, which showed that high creatinine levels correlate with a 3 to 5 times greater risk of death in STEMI patients [29, 30, 31]. Persistent high creatinine levels can indicate systemic congestion and fluid overload, leading to cardiac dysfunction and an increased risk of heart failure and arrhythmias. Monitoring renal function in post-PCI patients is essential to better tailor treatment strategies and reduce the risk of mortality.

GLT, a metabolic enzyme, also showed significant predictive value for MACE in STEMI patients in our study. While the relationship between GLT and cardiovascular diseases is still not fully understood, its role in metabolic regulation warrants further research to clarify its impact on adverse cardiac events.

Finally, PCT, an indicator of platelet function, was positively associated with MACE risk in this study, a finding consistent with the work of Reddy *et al*. [32]. Higher PCT levels suggest increased platelet aggregation, accelerating coronary plaque formation and an increased risk of cardiovascular disease. This may be due to the fact that elevated PCT reduces vasodilation and myocardial blood flow after PCI, contributing to further cardiac damage [33]. Additional large-scale studies are needed to further investigate the link between PCT and the risk of MACE in STEMI patients.

## 5. Limitations

This study utilized a retrospective research methodology. Therefore, the results may be affected by sample selection bias. There are numerous factors affecting the occurrence of MACE after PCI in patients with STEMI, and the present study may not have taken into account all the potential predictive variables, especially the relevant variables such as lifestyle and socioeconomic status, which have a significant impact on the risk of developing cardiovascular disease. Future studies will need to further incorporate these factors into the constructed model in order to more comprehensively assess the risk for adverse cardiovascular events in patients with an acute STEMI after a PCI. In addition, our model was constructed and validated based on a single dataset, which may limit the applicability and generalization of the model to other patient groups. Therefore, in order to better validate and optimize the predictive performance of the model, further larger sample, prospective, multicenter cohort studies will need to be conducted in the future.

## 6. Conclusions

In summary, in this study, a total of 7 important characteristic variables related to the occurrence of MACE after PCI in patients with an acute STEMI were screened based on ML algorithms and incorporated into an optimal logistic regression model, which can provide sufficient evidence-based support and methodological references for the early identification of high-risk groups of patients at risk for developing MACE after a PCI. This will help healthcare workers to more deeply understand the complex relationship between these indicators and the disease, and make targeted clinical scientific decisions, so as to improve more favorable patient prognoses and enhance long-term quality of life. The powerful predictive power of the logistic regression model in this study highlights the great potential of ML in the management and application of cardiovascular diseases. The advantage of such models lies in their ability to efficiently handle nonlinear relationships and a large number of interaction effects among data, yielding more accurate and reliable results, and can be extended, applied, and optimized to suit different clinical scenarios. However, to transform these models into practical tools in clinical practice, a series of factors will still need to be incorporated, including the quality and accessibility of data, the interpretive power of the models, and their integration with existing clinical workflows. In addition, with the rise of personalized medicine, the application of ML models in providing individualized treatment recommendations will need to be further explored and investigated in the future.

## Availability of Data and Materials

The datasets used and analyzed during the current study are available from the corresponding author on reasonable request.

## Appendix

See Table 5.

**Table 5.** **Variables and abbreviations**.

Variable	Full name of the variable	Type of variable	Unit of variability/Variable encode
Sex	sex	continuous variable	0 = men, 1 = women
Age	age	continuous variable	year
Killip	killip	categorical variable	1 = class I,
			2 = class II,
			3 = class III,
			4 = class IV
SP	systolic blood pressure	continuous variable	mmHg
BP	diastolic blood pressure	continuous variable	mmHg
HR	heart rate	continuous variable	times/min
Smoking	smoking	categorical variable	0 = NO, 1 = Yes
Lesion location	lesion location	categorical variable	0 = left trunk,
			1 = left anterior descending branch (LAD),
			2 = left revolving branch (LCX),
			3 = right coronary artery (RCA)
Number of vessels diseased	number of vessels diseased	categorical variable	/
Gensini	coronary artery lesion stenosis score	continuous variable	score
Thrombolysis	thrombolysis	categorical variable	0 = NO, 1 = Yes
Bivalirudin	bivalirudin	categorical variable	0 = NO, 1 = Yes
Trifluoroacetate salt	trifluoroacetate salt		
Number of stents	number of stents	categorical variable	/
WBC	white blood cell	continuous variable	10^9^/L
N	absolute neutrophil value	continuous variable	10^9^/L
L	lymphocytes absolute value	continuous variable	10^9^/L
M	absolute monocyte value	continuous variable	10^9^/L
RBC	red blood cell	continuous variable	10^12^/L
HB	hemoglobin	continuous variable	g/L
PLT	platelets count	continuous variable	10^9^/L
MPV	mean platelet volume	continuous variable	fL
PCW	platelet volume distribution width	continuous variable	fL
PCT	blood platelet count	continuous variable	%
GLU	glucose	continuous variable	mmol/L
BUN	blood urea nitrogen	continuous variable	mg/dL
CR	creatinine	continuous variable	umol/L
UA	uric acid	continuous variable	umol/L
Total protein	total protein	continuous variable	g/L
DBIL	direct bilirubin	continuous variable	umol/L
IBIL	indirect bilirubin	continuous variable	umol/L
GLT	glutamine transferase	continuous variable	U/L
GST	glutathione S-transferase	continuous variable	U/L
TG	triglyceride	continuous variable	mmol/L
TC	total cholesterol	continuous variable	mmol/L
HDL	high density lipoprotein	continuous variable	mmol/L
LDL	low density lipoprotein	continuous variable	mmol/L
VLDL	very low density lipoprotein	continuous variable	mmol/L
LVD	left ventricular internal diameter	continuous variable	mm
TDM	type 2 diabetes mellitus	categorical variable	0 = NO, 1 = Yes
HP	high blood pressure	categorical variable	0 = NO, 1 = Yes
